# Dentistry in Japan

**Published:** 1895-12

**Authors:** 


### EDITORIAL.

### Dentistry in Japan.

Japan is regarded as *one* of the most, if not the most, progressive nation of the East. While this is true of almost every thing else, it has not generally been known that dentistry has received much attention; but they have not been idle in respect to dentistry, as is shown by the fact that they have two dental journals, a large dental society and a dental college. "*The Shikagakukai Journal*" is the organ of the dental society and publishes the proceedings of that body in full. This journal was established about six years ago. The form is the same size of the REGISTER and each number contains about one hundred pages.

The other "*The Shikwa-igaku-sodan.*" "a medical journal devoted to the investigation of dental science."

The first number of this was issued October, 1895. It is the organ of, and is published by the Takayama Dental College in Tokio. It contains one hundred and twenty pages, and is published quarterly.

The dental society was organized in November, 1890. "The objects of the society is the improvement of the profession, and the exchange of dental knowledge."

"The meetings are held twice a month, when the members deliver speeches, open debate or answer questions on professional matters; and all these transactions are published in the monthly journal, which is distributed to the members. The total number of members is five hundred and twenty. They are composed of the following: 1st. Those who have passed the dental surgery public examination, which is exacted twice a year by the government; of this class of members there are two hundred and eighty-one. 2nd. The graduates of foreign dental colleges who are now actually engaged in the practice of the profession. 3d. Those who have certificates for the profession given from the government on account of their practice in the Japanese system of dentistry. 4th. The students of the art."

The college was founded in 1890, and is the only one in Japan. "It aims to promote the cause of dentistry, by treatting all matters in regard to it, practical as well as theoretical."

It is remarkable that all this should have been done within the last six years. All the agencies for development and growth put into active operation within that time. Our dental associations must bestir themselves or be outstripped by our Japanese friends.

